# *PTCH* mutations and deletions in patients with typical nevoid basal cell carcinoma syndrome and in patients with a suspected genetic predisposition to basal cell carcinoma: a French study

**DOI:** 10.1038/sj.bjc.6603303

**Published:** 2006-08-15

**Authors:** N Soufir, B Gerard, M Portela, A Brice, M Liboutet, P Saiag, V Descamps, D Kerob, P Wolkenstein, I Gorin, C Lebbe, N Dupin, B Crickx, N Basset-Seguin, B Grandchamp

**Affiliations:** 1 Laboratoire de Biochimie Hormonale et Génétique, Hôpital Bichat-Claude Bernard, AP-HP, Faculté de Médecine Paris VII, Paris, France; 2 Service de Dermatologie, Hôpital Saint-Louis, AP-HP, Faculté de Médecine Paris VII, Paris, France; 3 Service de Dermatologie, Hôpital Ambroise Paré, AP-HP, Faculté de Médecine Paris-Ile de France Ouest, Boulogne Billancourt, France; 4 Service de Dermatologie, Hôpital Bichat-Claude Bernard, Paris, AP-HP, Faculté de Médecine Paris VII, Paris, France; 5 Service de Dermatologie, Hôpital Henri Mondor, Créteil, AP-HP, Faculté de Médecine Paris XIII, Paris, France; 6 Service de Dermatologie, Hôpital Cochin-Tarnier, Paris, AP-HP, Faculté de Médecine Paris V, Paris, France

**Keywords:** PATCHED, NBCCS, multiple basal cell carcinoma, deletion

## Abstract

The patched (*PTCH*) mutation rate in nevoid basal cell carcinoma syndrome (NBCCS) reported in various studies ranges from 40 to 80%. However, few studies have investigated the role of *PTCH* in clinical conditions suggesting an inherited predisposition to basal cell carcinoma (BCC), although it has been suggested that *PTCH* polymorphisms could predispose to multiple BCC (MBCC). In this study, we therefore performed an exhaustive analysis of *PTCH* (mutations detection and deletion analysis) in 17 patients with the full complement of criteria for NBCCS (14 sporadic and three familial cases), and in 48 patients suspected of having a genetic predisposition to BCC (MBCC and/or age at diagnosis ⩽40 years and/or familial BCC). Eleven new germline alterations of the *PTCH* gene were characterised in 12 out of 17 patients harbouring the full complement of criteria for the syndrome (70%). These were frameshift mutations in five patients, nonsense mutations in five patients, a small inframe deletion in one patient, and a large germline deletion in another patient. Only one missense mutation (G774R) was found, and this was in a patient affected with MBCC, but without any other NBCCS criterion. We therefore suggest that patients harbouring the full complement of NBCCS criteria should as a priority be screened for *PTCH* mutations by sequencing, followed by a deletion analysis if no mutation is detected. In other clinical situations that suggest genetic predisposition to BCC, germline mutations of *PTCH* are not common.

Nevoid basal cell carcinoma syndrome (NBCCS, or Gorlin's syndrome) is an autosomal dominant syndrome predisposing to basal cell carcinomas (BCCs) and numerous developmental abnormalities (Gorlin, 2004). The prevalence is estimated at one per 57 000 (Evans *et al*, 1991); approximately 0.4% of all cases of BCC and 2% of BCC patients under 45 years of age are affected by NBCCS (Farndon *et al*, 1992).

NBCCS has been linked to germline mutations in the human homologue of the *Drosophila* segment polarity gene patched (*PTCH*) (Hahn *et al*, 1996; Johnson *et al*, 1996), and the rate of neomutation is high (Shanley *et al*, 1994). The *PTCH* mutation frequency in NBCCS patients reported varies considerably in the different studies, ranging from 40 to 80% (Kimonis *et al*, 2004; Marsh *et al*, 2005).

The *PTCH* gene consists of 23 exons, and encodes a 1447-amino-acid integral membrane protein, with 12 transmembrane regions, two extracellular loops, and a putative sterol-sensing domain. Most *PTCH* germline mutations are predicted to lead to premature truncation of the Ptc1 protein, and assumed to represent null *PTCH* alleles (Wicking and Bale, 1997), suggesting that many aspects of the phenotype apart from BCC result from haploinsufficiency. Tumours in NBCCS patients are likely to arise when the remaining *PTCH* allele is inactivated, which would be consistent with *PTCH* acting as a tumour suppressor gene (Gailani *et al*, 1992).

In addition, deletions of interstitial chromosome 9q have been identified in some NBCCS patients (Olivieri *et al*, 2003; Haniffa *et al*, 2004; Boonen *et al*, 2005).

One problem that arises is the possibility of a misdiagnosis of NBCCS, because of the complex phenotype of this syndrome. Various clinical and radiological criteria have been used to diagnose NBCCS; these are categorised as major and minor criteria. Nevoid basal cell carcinoma syndrome is considered to be certain when at least two of the four major criteria are present (multiple BCC (MBCCs), palmar and plantar epidermal pits, jaw keratocysts, and cerebral calcification) (Shanley *et al*, 1994). Patients may also display many other clinical features that are classified as minor criteria (Table 1) (Lo Muzio *et al*, 1999).

In addition to NBCCS, recent publications suggest that allelic variation of *PTCH* could also influence susceptibility to BCC (Strange *et al*, 2004a, 2004b; Asplund *et al*, 2005). In particular, some *PTCH* haplotypes, including polymorphisms in exon 23 (c.3944C), intron 15 (G^2560+9^), or exon 12 (c.1686C), seem to have a potentially protective effect against BCC (Strange *et al*, 2004a, 2004b).

The goal of this study was to search for *PTCH* germline abnormalities both in patients harbouring all the criteria for NBCCS and in those clinically suspected of having a genetic predisposition towards BCC (MBCC and/or BCC while under 40 years of age and/or familial BCC).

## PATIENTS AND METHODS

### Selection of patients

This study was performed from 2003 to January 2005. Patients were enrolled at the Saint Louis (60%), Ambroise Paré (25%), Bichat-Claude Bernard (5%), Tarnier (5%), and Henri Mondor (5%) hospitals, all of which are located in or near the city of Paris (France). Sixty-five patients were prospectively enrolled in the study, 10% of whom were newly diagnosed cases. Two different categories of patients were studied:

1. Patients affected by the typical NBCCS (17 index cases: three familial and 14 sporadic) who displayed at least two of the major criteria (MBCC, palmo-plantar pits, cerebral calcifications, odontogenic keratocysts) or one major criterion plus at least two minor criteria as defined by Shanley *et al* (1994). In addition, seven additional NBCCS patients from the three enrolled families were also studied.
2. Patients strongly suspected of having a genetic predisposition towards BCC (48 cases), characterised by either (i) MBCCs (35 cases), defined as the presence of at least two BCCs in the same patient confirmed by pathology reports, and/or (ii) BCC in patients under 40 years of age (28 cases) and/or (iii) familial BCC (10 cases), defined as the presence of at least two BCC cases in first- or second-degree relatives (all cases confirmed by pathology reports). For familial BCC cases, only the proband was enrolled. To exclude the presence of NBCCS features in this ‘BCC-predisposed’ non-NBCCS group, a careful clinical exam was realised to search for BCC, pits on palm and soles, facial, ocular, and limbs abnormalities. In addition, dental and crane X-rays were also performed in order to verify the absence of odontogenic keratocysts and intracranial calcifications.

Written informed consent, agreeing to peripheral blood sampling and genetic analysis, was obtained from each patient enrolled in the study. Genomic DNA was isolated from peripheral blood leucocytes of all the participants by routine methods (Miller *et al*, 1988).

### *PTCH* sequencing

The 23 exons of the *PTCH* coding sequence were amplified using 23 primer pairs (Table 2). PCR conditions included 35 denaturing cycles at 95°C for 30 s, annealing at 60°C for 30 s, elongation at 72°C for 45 s for exons 1 and 4; and 35 denaturing cycles at 96°C for 30 s, annealing at 63°C for 30 s, elongation at 72°C for 1 min for exons 5–23. Sequence analysis was performed on an ABI-Prism 3100 automated DNA sequencer using 10 ng PCR purified products and Big-Dye Terminator Cycle Sequencing kits (Perkin Elmer, Courtaboeuf cedex, France), according to the manufacturer's instructions. The functionality of the nonsynonymous variant was predicted using the *Polyphen* and SIFT informatics program (http://tux.EMBL-Heidelberg.DE/ramensky/; http://blocks.fhcrc.org/sift/SIFT_seq_submit2.html).

### *PTCH* deletion analysis

#### Real-time quantitative PCR

Real-time quantitative PCR was performed using SYBR Green I dye as a fluorescent signal. This dye binds specifically to the minor groove of double-stranded DNA, making it possible to detect PCR product formation (Ginzinger, 2002).

In order to examine both ends of *PTCH*, two targets were initially chosen on *PTCH* exons 1 and 23, and then, to extend the analysis, two other *PTCH* targets on exons 4 and 15, respectively, were also examined (Table 2). Two single-copy sequences were used as reference sequences: MYH9, mapping at 22q13.1, and Rb, mapping at 13q. Five microlitres of DNA was added to the PCR reaction mixture containing 1 × SYBR Green buffer (Applied Biosystems, Courtaboeuf cedex, France), 300 nM forward and reverse primers, 5 mM MgCl_2_ (3 mM for 8q11 SST), 200 *μ*M dNTP, and 0.6 U of AmpliTaq Gold (Applied Biosystems) in a final volume of 25 *μ*l.

Each series of PCR reactions included two negative controls, containing water in place of DNA, and a five-point standard curve. The standard curve was plotted using serial dilutions of normal PBMC in Tris (10 mM)–EDTA (1 mM) buffer, ranging from 10 to 0.02 ng *μ*l^−1^ (corresponding to 50–0.1 ng of DNA analysed per well). The same dilutions were used for all targets and reference sequences. PCR was performed on the ABI PRISM 7700 Sequence detector system (Applied Biosystems). All analyses were performed in duplicate. The PCR amplification profile was as follows: initial denaturing at 95°C for 10 min, followed by 40 denaturing cycles at 95°C for 10 s, and a combined annealing and extension step at 65°C for 1 min. Detection of the fluorescent product was carried out at the end of the extension period. To confirm amplification specificity, the PCR products from each primer pair were subjected to a melting curve analysis, and subsequent agarose gel electrophoresis. The concentration of each gene was calculated based on the appropriate calibration curve. Relative copy numbers of *PTCH* were then obtained by calculating the ratio of the result obtained for each target to the MYH9 and Rb value. The normalised ratio of each target on MYH9 and Rb was expected to be close to 1, if no deletion had occurred.

### Microsatellite analysis

Two microsatellite markers were studied: (i) a CGG repeat localised in the 5′UTR, which was genotyped by sequencing *PTCH* exon 1, and (ii) a CA repeat localised in intron 2 (Aboulkassim *et al*, 2003), which was genotyped by migration of the fluorescent-labelled PCR product on a 310 Genetic Analyzer (Applied Biosystems).

### Multiplex ligation-dependent probe amplification

*PTCH* deletion was also investigated by multiplex ligation-dependent probe amplification (MLPA), a quantitative, multiplex PCR method, as described previously (Gille *et al*, 2002). Multiplex ligation-dependent probe amplification was used to determine the relative copy number of each of the 23 *PTCH* exons, and was performed using an available commercial kit (SALSA MLPA KIT P067 PTCH, mrc-Holland).

## RESULTS

Seventeen patients were considered to have NBCCS, on the basis of the presence of two major criteria, or of one major criterion plus two or more minor criteria. Three were nonrelated familial cases, and 14 were sporadic cases. The three families had, respectively, four, three, and two NBCCS patients, all first-degree related. Thirteen patients had two or more major NBCCS criteria (four patients with two major criteria, seven patients with three major criteria, and two patients with all four criteria). Four NBCCS patients had only one major criterion plus two, four or five minor criteria. The frequencies of the major criteria were as follows: MBCC (88%), palmo-plantar pits (78%), odontogenic keratocysts (70%), and cerebral calcifications (57%). The most frequent ‘minor’ criteria were macrocephaly (70%), epidermal cysts (60%), scoliosis (60%), hypertelorism (50%), and strabism (36%). The median age at the first BCC in this NBCCS group was 27 years.

Forty-eight patients suspected of being predisposed to BCC were characterised by either (i) the occurrence of MBCC (35 cases) and/or (ii) the occurrence of BCC before the age of 40 years (28 cases), and/or (iii) the presence of familial BCC (10 cases), defined as the presence of at least two BCC cases in first- or second-degree relatives. The median age at the first BCC in this group was 42 years.

*PTCH* mutations were identified in 12 out of 17 patients harbouring the full complement of criteria for NBCCS. These were frameshift mutations in five patients, nonsense mutations in five patients, and one in frame deletion in one patient (see Table 3). An identical, nonsense mutation, W129X, was characterised in two unrelated patients. *PTCH* mutations were detected in all three familial cases, and were shown to segregate with the disease in the families, as they were detected in all the seven relatives affected by NBCCS (Table 3).

In addition, a large germline deletion was detected in another typical NBCCS patient. Quantitative PCR analysis showed that three of the four exons examined (4, 15, 23) were deleted, whereas the first exon was not. As the patient was heterozygous for a microsatellite localised in intron 2, this means that the deletion must begin after exon 2 of *PTCH*. These results were confirmed by MLPA, with a 50% reduction in signal intensity from exons 5 to 23, whereas exon 3 was normal. As the MLPA kit does not explore exon 4, both results are concordant and show the presence of a large *PTCH* deletion including exons 4–23. *PTCH* deletions were also looked for in the five remaining NBCCS patients who did not harbour any *PTCH* mutation, but none was found. To summarise, therefore, germline mutations or deletions of *PTCH* were present in 70% of NBCCS patients.

In contrast, in the BCC group without any other criterion for NBCCS, only one missense variant, G774R, was found in a patient affected with MBCC. This patient had five different BCCs, all localised in the head and neck region, the first BCC being diagnosed at the age of 46 years. This variant localised in the putative fourth extracellular domain, and is predicted to be damaging by the SNP prediction programs Polyphen and SIFT (http://tux.embl-heidelberg.de/ramensky/; http://blocks.fhcrc.org/sift/SIFT.html). No large deletions of *PTCH* were observed by real-time PCR or MLPA in the remaining patients with a suspected genetic predisposition to BCC.

## DISCUSSION

In this study, we identified *PTCH* mutations or deletions in 12 out of 17 patients with NBCCS (70%). As far as we know, only one study has been performed in the French population (Boutet *et al*, 2003). Of the 11 mutations identified in NBCCS patients, 10 resulted in truncation of the *PTCH* protein owing to frameshifts or nonsense mutations. This is consistent with the finding that most (86%) mutations lead to premature termination of the protein (Wicking *et al*, 1997; Fujii *et al*, 2003).

Previously, *PTCH* mutations have been found in 40–80% of NBCCS patients (Chidambaram *et al*, 1996; Wicking *et al*, 1997; Boutet *et al*, 2003). Although our group is quite small, the exhaustive screening for *PTCH* exons and flanking intronic regions by direct sequencing and deletion analysis may have increased the mutation detection rate.

We identified a large *PTCH* deletion in a patient harbouring the typical signs of NBCCS. In all, five patients that share NBCCS features were previously been reported to carry an interstitial chromosome 9q deletion identified by cytogenetic analysis (Shimkets *et al*, 1996; Sasaki *et al*, 2000; Haniffa *et al*, 2004; Midro *et al*, 2004). This indicates that large *PTCH* deletions are not a rare mechanism of *PTCH* inactivation, and this possibility should be investigated if no *PTCH* mutation is detected.

Despite the exhaustive analysis, no *PTCH* mutation or large deletion was found in five of the NBCCS patients. This is likely to be due to the existence of mutations outside the regions analysed possibly in introns or regulatory elements. An alternative hypothesis could be the presence of a somatic mosaicism, or the existence of mutations in another gene implicated in the sonic hedgehog pathway, as has been shown to occur in sporadic BCC (Reifenberger *et al*, 1998; Xie *et al*, 1998).

In the group of BCC patients without any other NBCCS criterion, only one missense mutation (G774R) was found in a patient with MBCC without any other NBCCS criteria (in particular, this patient had a normal head circumference, no facial or ocular abnormalities, and the chest and crane X-rays did not show any skeletal abnormality or intracranial calcification). Unfortunately, segregation could not be assessed because his parents were deceased. Therefore, the significance of this amino-acid substitution will not become completely clear until a functional analysis is performed. However, this could be a causative mutation, as (i) it is predicted to be damaging by two bioinformatic programs *Polyphen* and *SIFT* and (ii) it was not reported in any previous study or in the NCBI SNP database. On the other hand, we cannot exclude the possibility that this could be a rare polymorphism.

We did not found any other *PTCH* mutation in this group (*P*<0.0001), which indicates that when other NBCCS criteria are absent, *PTCH* mutations are rarely involved in predisposition to BCC. Nevertheless, it remains possible that *PTCH* polymorphisms located outside the coding sequence or intron–exon junctions could influence BCC susceptibility, as has been suggested by recent publications (Strange *et al*, 2004a, 2004b; Asplund *et al*, 2005).

In conclusion, germline abnormalities (mutations and deletions) of *PTCH* are very predominantly observed in patients with the full criteria for NBCCS. We therefore suggest that patients harbouring the full complement of NBCCS criteria should, as a priority, be screened for *PTCH* mutations by sequencing, followed by a deletion analysis if no mutation is detected. The finding of a *PTCH* mutation confirms the clinical diagnosis of NBCCS, therefore validating the clinical and radiological diagnostic criteria of this syndrome. The molecular confirmation of NBCCS diagnosis permits a better clinical monitoring (in particular, dermatological), a choice of the rational therapeutic (e.g. avoiding radiotherapy for treatment of BCCs). Moreover, it makes it possible to carry out a genetic council in the families concerned, and to offer the possibility of antenatal diagnosis if the families wish it.

## Figures and Tables

**Table 1:** NBCCS minor criteria

**NBCCS minor criteria**
Congenital skeletal anomaly: bifid, fused, splayed, or missing rib, bifid, wedged or fused vertebra, cyphoscoliosis, brachydactyly, short fourth metacarpal, short thumb terminal phalanx
Macrocephaly, frontal bossing, prognathism
Congenital mouth malformation: cleft lip or palate, coarse face, polydactyly
Eye anomaly: strabismus, hyperthelorism, cataract, coloboma, microphtalmia
Cardiac or ovarian fibroma
Medulloblastoma
Lymphomesenteric cysts, congenital lung cyst
Mental retardation

NBCCS=nevoid basal cell carcinoma syndrome.

**Table 2:** *PTCH* PCR primers

**Exon number**	**Primer name**	**Primer sequence (5′ → 3′)**
*Sequencing primers*		
1	PTCH Ex 01F	TGG AAG GCG CAG GGT CTG ACT
	PTCH Ex 01 R	CGA TCC CAA AGA GTT AGA GGA
2	PTCH Ex 02F	CTG CGG CCC GGC TTT ATG AC
	PTCH Ex 02R	GTG TGC GCT GGC GAA TAT CTC TAT C
3	PTCH Ex 03F	ACT GCT CAC ACA TCA GCC AGT CTC AT
	PTCH Ex 03R	GCA TTT CCA GGG CAA CTT CAT TTA CTA
4–5	PTCH Ex 04–05F	GCT GGG TCT CTA CTT GGC AAA AGC
	PTCH Ex 04–05R	CCC GAC TAT TCA CTC AAA AAA TGC ACA
6	PTCH Ex 06F	ATT TGT TTT GAT GCC AGA GTC CCA GA
	PTCH Ex 06R	GGC TAA TGG GAG GTG TAT GGC AAA TC
7	PTCH Ex 07F	AAG ATT TGC CAT ACA CCT CCC ATT AGC
	PTCH Ex 07R	AAT TCC CCA CAA GGT GCT TTT TCA A
8	PTCH Ex 08F	GGA AAC ATG TGC TCA CAG AGA AGG AAA
	PTCH Ex 08R	TCC CAT CAA GTT CCC AGA ATT GCA
9	PTCH Ex 09F	CCC TGC CCT GGA ATC ACG TAG AAC
	PTCH Ex 09R	GAA GCA GGA GCA GTC ATG GAA AAG TAA
10	PTCH Ex 10F	TTT GCC GTT TGC CTA CCT TTG ACT C
	PTCH Ex 10R	CGG TGA GAA GGA CAC ACA GCA CAC
11	PTCH Ex 11F	AGG TGC TGG TGG CAG AGT CCT AAC TA
	PTCH Ex 11R	GCA GCC AGT GAC ACA TCA TCT GAC AT
12	PTCH Ex 12F	CTG CCA CGT ATC TGC TCA CAC AGT C
	PTCH Ex 12R	CAC CCA GTT AAA CAG AGC CTC AAA CAC
13	PTCH Ex 13F	CAC GGT TTC AAA TGC TTC AAG AGG A
	PTCH Ex 13R	CAA ACC CCG TTA CCC ACA TTC CTT
14	PTCH Ex 14F	CAG GCG ATG AAC CAG GTG ATG TTA T
	PTCH Ex 14R	GAA GCA ATC TGA TGA ACT CCA AAG GTT
15	PTCH Ex 15F	TTG TCC AGG AAG AGT CAG TGG TGC TC
	PTCH Ex 15R	GTT GAA GCT GAA CAC GCA AAA GAC C
16	PTCH Ex 16F	CCC TGC CCT GCT CAG TCT CCT C
	PTCH Ex 16R	CTG GCA TGA GGT CAC ACA ATT AGC TG
17	PTCH Ex 17F	GCC AGT GAT TGC ATC CTC CGA TAA
	PTCH Ex 17R	CCA TTA CAC ATC CTC GTC TCC CAG AG
18	PTCH Ex 18F	CCT CAC AAA GAA TGA CTG CTG GAA GAT
	PTCH Ex 18R	CCA GAG GCC CAG ACA TAA ACA AAA CTT
19	PTCH Ex 19F	AAG GTT CCC ACT TGG AGA CAA ACA GAG
	PTCH Ex 19R	TGA ATT AGG CAG TAA AGG CAG TGT CCA
20	PTCH Ex 20F	TAC GTC AAC ACC AAA TAT GAC CCA GTG
	PTCH Ex 20R	TCT GCC TCA GCC TCC CAA GTA GC
21	PTCH Ex 21F	TGA ATG TGA ACT GCG GTT GGA TAA CA
	PTCH Ex 21R	CCA GTA CAC CGA AGA GGA AAA CAG ACA
22	PTCH Ex 22F	CCC CTG AAA AAT ACC GTG CTT TGA G
	PTCH Ex 22R	ATC TGC CTG TGT GAT GTG CTG CTC
23	PTCH Ex 23F	GGG TTG ACT GAG TCT TTG GTG AAA CC
	PTCH Ex 23R	TTG TCC TCC TCT TTG CCT GGC TCT A
		
*Quantitative PCR primer*		
	PTCHqex1F	CCA AAG AGT TAG AGG AGG GAA GAG AAA GT
	PTCHqex1R	CTA TCT GCA CCG GCC CAG CTA C
	PTCHqex4F	GCT GGG TCT CTA CTT GGC AAA AGC
	PTCHqex4R	TTT CCA CTG CCT AAT AAA ATG AAA AGC
	PTCHqex15F	AAG AAA ACA AAC AGC TTC CCA AAA TGT
	PTCHqex15R	GTT GAA GCT GAA CAC GCA AAA GAC C
	PTCHqex23F	TCC AGC CAG CCG TGT CAG AGA
	PTCHqex23R	TTC CAC CCA CAA AAG AAA AGC CTG T

NBCCS=nevoid basal cell carcinoma syndrome; PTCH=patched.

**Table 3:** *PTCH* mutations in NBCCS and MBCC patients

**Patient**	**Diagnosis**	**Exon**	***PTCH* mutation**	**Effect on protein**	**Familial**	**Segregation**
B249	NBCCS	4–23	del	Truncated	−	NA
P270	NBCCS	2	c.385 G>A	W129X	−	NA
B530	NBCCS	15	c.2443–2461 del 18	p. I815N Del (Q816, H817, L818, L819, Y820, D821)	+	Yes, four cases
B344	NBCCS	15	c.2450 T>A	L818X	−	NA
B370	NBCCS	17	c.2712 C>T	Q905X	−	ND
B395	NBCCS	6	c.922 delG	p. A308PfsX323	+	Yes, two cases
B401	NBCCS	18	c. 2962 dup TT	p. V988LfsX995	+	Yes, one case
B419	NBCCS	18	c.3053 G>A	W1018X	−	NA
B420	NBCCS	17	c. 2743 ins CATCATT	p. N915Hins7fsX917	−	NA
P433	NBCCS	2	c.260–265 delTTTA	p. F88Ndel4fsX116	−	NA
B484	NBCCS	2	c.291 insA	p. N97KfsX139	−	NA
B519	NBCCS	2	c.385 G>A	W129X	−	NA
P345	MBCC	15	c.2320 G>A	G774R	−	NA

PCTH mutations are described using the nomenclature system for human gene mutations (den Dunnen and Antonarakis, 2001).

MBCC=multiple basal cell carcinoma; NA=not applicable; NBCCS=nevoid basal cell carcinoma syndrome.
